# Internet Use and Better-Informed Divorce in China

**DOI:** 10.3390/bs13020177

**Published:** 2023-02-15

**Authors:** Jindian Liu, Ning Neil Yu, Mingwang Cheng, Chunyan Wu

**Affiliations:** 1 College of Economics & Management, Northwest A&F University, Xianyang 712100, China; 2 Institute for Social and Economic Research, Nanjing Audit University, Nanjing 210017, China; 3 School of Economics and Management, Tongji University, Shanghai 200092, China; 4 Institute for Higher Education, Northwest A&F University, Xianyang 712100, China

**Keywords:** Internet use, Internet penetration, divorce, information access, mandatory cooling-off period

## Abstract

China has witnessed a rapid expansion in Internet penetration in recent years, with profound impacts on people’s family life and marital relationships. This paper aims to examine the causal effects and functionary of information access through Internet on marital stability. This paper identifies a robust association between Internet use and increasing divorce rates in China by using nationally representative, individual-level survey data and province-level aggregate data. Various regression techniques and specifications demonstrated the statistical and economic significance of the association. Given the ever-expanding role of the Internet and the serious consequences of divorce on families and society, it is imperative that we study the underlying mechanisms as the first step toward socially responsible policymaking. Our analysis revealed a significant mediating effect of the self-reported importance of Internet information acquisition, the frequency of chatting with online friends, the frequency of meeting with online friends, and the intensity of Internet use. These findings are consistent with the theory that the increase in divorce decisions is due to better information access and is, therefore, rational and that policies such as a mandatory cooling-off period for divorce may lower societal welfare. We also conducted a series of heterogeneity analyses that showed, among other findings, that the Internet effect is stronger for women.

## 1. Introduction

**Figure 1:**
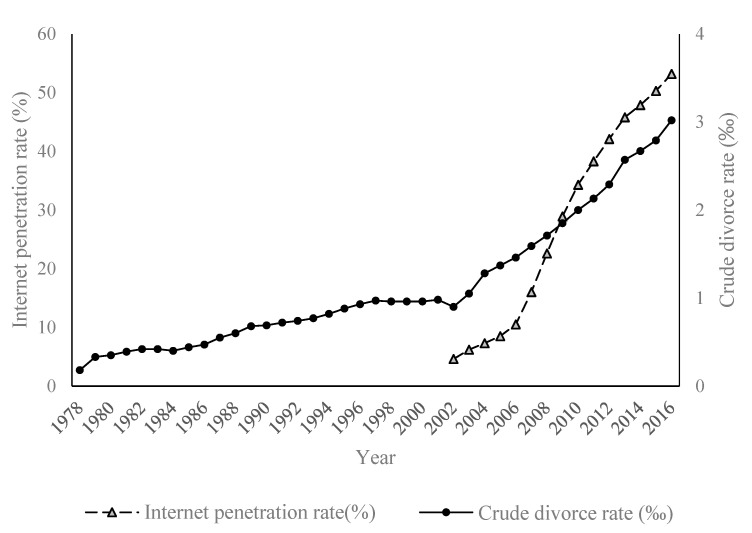
Trends in IPR and CDRs in China.

A sharp rise in crude divorce rates (CDRs) has been witnessed in the past decade, as well as a rise in the Internet penetration rate (IPR) across provinces in China. Despite China’s long adherence to traditional marriage notions and its viewing divorce as a stigma, the country’s divorce rates have significantly increased since 2002. Figure 1 shows that, before 2002, CDRs were less than one in a thousand, and the growth rate was slow. Divorce rates, however, demonstrated an obvious turning point in 2002, rising from 0.90 per thousand in 2002 to 3.02 per thousand in 2016. This leads us to ponder the shocks that have affected China’s marital stability since 2002.

As shown in Figure 1, since 2002, the Internet has rapidly penetrated Chinese daily life. The IPR in China has increased from 4.60% in 2002 to 53.00% in 2016. As of June 2017, the number of Chinese Internet users reached 751 million, accounting for one-fifth of the total number of Internet users in the world. The rapid popularization of broadband and mobile Internet has had profound implications on the political, economic, and cultural life of society [1,2,3], including social interactions and marital relationships [4]. Liu et al. (2020) found that China is experiencing an Internet-driven sexual revolution, which has accelerated the disintegration of traditional concepts of sex and marriage [5]. In fact, from 2000 to 2015, the incidence of extramarital sex among married adults aged 20 to 59 almost tripled [6], threatening marital stability. In this paper, we focused on the convergence of the IPR and CDRs to study whether the IPR is a potential factor that has increased divorce rates and the mechanism through which it affects marital stability.

The determinants of divorce have been well studied from social and economic perspectives, including the unemployment rate [7], house prices [8], population mobility [9], female labor force participation [10], and technological change [11]. Although a few studies have focused on the relationship between broadband Internet and the divorce rate [12], the impact and mechanism of the Internet’s influence on marital stability needs to be clarified. This paper aims to address this gap by investigating the impact and underlying mechanism of Internet use on marital stability through the use of national representative data and to further examine the effect of the IPR on CDRs, using provincial panel data from 2005 to 2016.

The steadily increasing rate of divorce has sparked great concern among some policymakers in China. They view divorce as a threat to economic and social stability and development. Driven by this view, China introduced a mandatory 30-day cooling-off period for divorce in the newly revised Civil Code, which came into effect on 1 January 2021. Before the cooling-off period policy was implemented, couples could complete the divorce procedures on the same day with valid documents, without unnecessary waiting. Since the implementation of the cooling-off period policy, couples who file for divorce must wait 30 days, during which time either party can withdraw the application. They must apply again at the end of the 30-day period to end the marriage. If no application is made, the application for divorce registration shall be deemed withdrawn. The implementation of the mandatory cooling-off period policy has, indeed, played a role in reducing the number of divorces. According to statistics from the Ministry of Civil Affairs of the People’s Republic of China, 296,000 people registered for divorce in the first quarter of 2021, representing a decline of 72 percent from 1.06 million in the fourth quarter of 2020 (http://www.mca.gov.cn/article/sj/tjjb/qgsj/ (accessed on 20 November 2022)).

Because scholars have not yet reached a consensus on the negative effects of divorce, however, we need to be prudent in judging whether divorce is beneficial or harmful to personal happiness. Extensive studies have documented the beneficial effects of marriage and the negative effects of divorce on personal health and well-being [13,14,15]. For instance, Holden and Smock (1991) argue that divorce can significantly affect the well-being of the adults and children concerned [16]. Adults and children from divorced families score lower than their counterparts in intact families on several indicators of well-being. Nevertheless, the role of marriage quality in the impact of marriage and divorce on personal well-being has not been fully studied [17]. It is apparent that the quality of marriage varies among married couples and that dissolving a problematic marriage relationship can be a solution rather than a problem. For women who suffer from an unfortunate marriage, divorce will help to improve their welfare. Some scholars believe that extricating oneself from an unfortunate marriage relationship may reduce women’s risk of depression, anxiety, and suicide [18].

In addition, the setting of the divorce cooling-off period is designed to reduce the rise in the divorce rate. It assumes that divorce behavior is an irrational decision made with incomplete information. By facilitating people’s access to information, however, the Internet reduces the stigma of divorce, improves people’s chances of searching for alternative partners, and gives individuals in unhappy marriages the opportunity to make new choices. If some originally happy marriages break down due to the threat of the Internet, it may result in the loss of welfare for both spouses. If Internet access allows some unfortunate marriages to break down, the welfare of the couple may improve. This is especially true for Chinese women. Because a sexual double standard still has a certain influence in China, the social and psychological pressures brought by divorce to women are much more severe than to men [19]. This makes women prefer to endure unhappy marriages and to not divorce.

If Internet access can provide emotional support and channels for finding alternative partners, women who endure an unfortunate marriage will be better off seeking a divorce. This is consistent with the “rational actors” perspective, which holds that Internet users will weigh the benefits and costs, such as a problematic relationship, and make the most advantageous choice [20]. Of note, the suicide risk for rural women in China is significantly higher than that for men [21]. Research has found that pressure from a family or husband increases the risk of female suicide and that an increase in women’s freedom to divorce serves as a protective factor [22].

According to this proposition, although some Internet users use the Internet as a tool to obtain emotional support and find potential partners, which threatens their current marriage relationship, we cannot infer that they are not aware of the risks of these behaviors to their current marriage and that, perhaps, these behaviors may better meet their needs and goals. For example, women in an unhappy marriage may seek emotional support and even meet new partners through the Internet to compensate for the lack of satisfaction with their emotional needs in their marriage. Therefore, divorce is a more rational decision that is made after obtaining sufficient information and social support, rather than an irrational decision made under the circumstances of insufficient information or impulsiveness. The introduction of a mandatory cooling-off period for divorce may discourage the early termination of an unhappy relationship, thereby lowering societal welfare.

Compared with the extant literature, this research makes four contributions. First, this study contributes to the literature on the determinants of marriage stability from the perspective of the technological development of the information industry. This is the first study, using nationally representative and individual-level data, to explore the impact of the Internet on marriage stability in the context of a Confucian culture. This paper investigated the association between Internet access and marital stability based on China Family Panel Studies (CFPS) data from 2010 to 2016. The results revealed that Internet access is another risk factor for marriage stability. The provincial study further examined the impact of the IPR on CDRs based on provincial panel data from 2005 to 2016. The results indicated a positive correlation between the IPR and CDRs. The provincial- and individual-level studies were consistent.

Second, this paper not only demonstrated the positive correlation between Internet access and marital stability, but also made causal inferences. For the individual-level study, we addressed the potential self-selection problem using the propensity score-matching method. The estimated results under four matching methods—nearest neighbor, radius, kernel, and local linear matching—were all significantly positive, which indicated that Internet access is positively correlated with the risk of divorce. For the provincial-level study, the independent variable regression results indicated that a one-percent increase in the IPR is associated with a 23.90 percent increase in CDRs.

Finally, by using a mediation effect model, we shed light on the mechanism through which Internet access affects marriage stability. The results indicated that the effect of Internet access on divorce risk occurs through the importance of Internet information acquisition, the frequency of chatting with online friends, the frequency of meeting in person with online friends, and the intensity of Internet use. The mediation effect model revealed that the above four mediators accounted for 48.74%, 35.70%, 8.06%, and 44.39%, respectively, of the total effect.

The remainder of the paper is organized as follows: Section 2 provides a systematic literature review on the influence of Internet use on marital stability and proposes four research hypotheses. Section 3 presents the methodology, variable settings, datasets, and some descriptive analyses. Section 4 contains an empirical examination of the impact and mechanism of Internet use on divorce risk from a micro perspective. The positive association between the IPR and CDRs is verified in Section 5. Section 6 provides a discussion and the conclusions of the study.

## 2. Background and Hypotheses

The driving forces and motivations of divorce have been widely studied. The marriage-matching theory proposed by family economics posits that the marriage-matching process is essentially the same as the job-search process in the labor market [23]. The stability of the marriage market means that marriage matching is in equilibrium, and divorce indicates that the equilibrium is broken [24]. Divorce occurs when one or both partners believe that the expected benefits of divorce or remarriage are higher than the existing marital status [25]. Bergner and Bridges (2002) found that a long-term and stable marriage relationship includes the following eight characteristics: (a) investment in the well-being of the beloved, (b) respect, (c) admiration, (d) sexual desire, (e) intimacy, (f) commitment, (g) exclusivity, and (h) understanding [26]. When one or more of the above characteristics are destroyed, the marital stability will decline. Ruiz (2008) identified the subjective motivations (such as not feeling loved) and objective motivations (such as violence) of divorce [27]. Based on theoretical analyses, empirical studies have found that changes in external economic and social conditions, including famine, income changes, female labor market participation, and policy intervention projects, are important factors that affect marriage dissolution [28,29]. The potential impact of technological advances represented by the IPR on marital stability, however, has not been fully studied. This study aims to bridge this gap.

The Internet plays an important role in disseminating modern marriage norms, decreasing the search cost, expanding social networks, and impinging on communication time between spouses. Considering the above factors, some scholars have empirically analyzed the impact of the Internet on marital stability and found that Internet use has a certain deleterious impact. Murray (2020) found that Internet access had a significant negative impact on marriage stability in rural U.S. counties [30]. Valenzuela et al. (2014), using interstate sample data from the United States, argued that Facebook penetration is one of the driving factors for the rise in divorce rates [20]. Although some evidence indicates that the Internet has certain negative implications for marriage stability, to the best of our knowledge, the mechanism through which this impact works has not been well established. Based mainly on family economics and related divorce studies, we offer four explanations for the mechanism by which the Internet affects marital stability.

### 2.1. Spreading the Modern Marriage Concept

In a feudal society, the norms of which have survived, divorce was stigmatized and people felt that they should make do in an unhappy marriage. The IPR, however, is accelerating the disintegration of such traditional concepts, and the concept of modern marriage, which emphasizes love and happiness, has achieved mainstream status. Social learning theory emphasizes the importance of observing and modeling the behaviors, attitudes, and emotional reactions of others and focuses on the interaction between the individual and the environment to understand certain aspects of behaviors [31]. In this regard, there is a noticeable demonstration effect on individuals from the behaviors, attitudes, and emotions of family members, peers, and even strangers.

As a new type of media, social networks have profoundly altered the landscape of people’s social interactions and have provided a platform through which strangers can be connected by shared interests, political views, or activities [32]. Due to the anonymity and virtual aspects of social network services, diverse marriage conceptions that deviate from traditional notions have diffused rapidly. As argued by Manago et al. (2012) and Sorokowski et al. (2015), online services are accelerating the change in people’s self-awareness and conception of life, and perceptions regarding marriage may be altered by continuous exposure to modern marriage notions [33,34]. The Internet has led society to become open and tolerant with regard to the concept of marriage [35]. Instead of feeling ashamed of divorce, people advocate the termination of unhappy marriages, which eventually reduces the social cost and psychological pressure of divorce [14]. Accordingly, we propose the first hypothesis:

**Hypothesis** **1.** *Through accelerating the spread of the concept of modern marriage and reducing the expected cost of divorce, Internet use leads to a rise in divorce rates.*

### 2.2. Lowering the Search Cost for Alternative Marital Partners

The Internet plays an important role in the search for alternative marriage partners, reducing the cost of finding an alternative partner after divorce, and even for finding an extramarital partner [36]. The probability of divorce tends to rise as the cost of divorce declines. According to bargaining theory, marriage is a long-term contract between two individuals, including a series of agreements, such as mutual companionship, child rearing, and family income acquisition [28,37]. The signing and termination of this contract are the result of the decision making of individuals to maximize their utility under certain constraints. If the expected return of marriage is lower than the loss from divorce, the marriage will be dissolved. In fact, Kendall (2011) argues that when people have more information about “potential alternate marriage partners” and can easily find new partners after divorce, the expected benefits from a new marriage may exceed the cost of breaking up the old one, thus increasing divorce rates [38]. Social network sites targeted at making friends online and sharing communication, such as Facebook and WeChat, have a series of unique functions (e.g., WeChat’s shake, people nearby) to help people reduce search costs, thus leading to a decline in the search cost of finding alternative partners [39]. Therefore, we propose the second hypothesis:

**Hypothesis** **2.** *Internet use leads to higher divorce risk by lowering the search cost for finding alternative marital partners.*

### 2.3. Expanding the Range of Alternative Marital Partners

The exit-threat bargaining model proposed by Manser and Brown (1980) and McElroy and Horney (1981) emphasizes the bargaining power of each party within the marriage, as affected by each other’s best option outside the marriage [37,40]. The Internet has changed the opportunities provided to couples outside of marriage, thus altering their bargaining power and, ultimately, disrupting the equilibrium of marriage [29]. The Internet has greatly expanded people’s social networks, thus significantly broadening the range of potential marital partner choices. The more alternative spouses, the more vulnerable the stability of the current marriage [41]. In traditional society, interpersonal communication was severely restricted by kinship and geographical scope. Since entering the information age, the advancement of smart devices and the popularity of instant messaging applications have dramatically expanded people’s social networks, both geographically and in complexity [42]. Kraut et al. (2002) argue that the Internet provides a way for people who are geographically distant to communicate and develop relationships [43]. Hjorth et al. (2014) believe that social media networks, such as QQ (Tencent, Shenzhen, China), WeChat (Tencent, Shenzhen, China), and Weibo (Sina, Beijing, China), have become the main means for Chinese people to maintain old relationships as well as develop new ones [44]. By increasing the number of available potential partners, Internet use increases the divorce risk. Therefore, we propose the third hypothesis:

**Hypothesis** **3.** *Internet use exacerbates divorce risk by expanding the range of alternative marital partners.*

### 2.4. Impingement of Communication between Husband and Wife

The Internet is widely used in work, study, entertainment, and life in general. The excessive use of the Internet may cause problems with the maintenance of interpersonal relationships [45,46,47,48]. Online communication often replaces face-to-face communication, crowding out the time people share in real life. Excessive time spent on the Internet substantially impinges on the interactions between a husband and wife, leading to seemingly irreconcilable differences and the breakdown of the marriage. It is commonplace in news reports that many young couples are immersed in playing with mobile Internet devices without communicating with each other (http://news.sina.com.cn/o/2015-05-06/145931800367.shtml (accessed on 25 November 2022)). To a certain extent, mobile Internet devices serve as a substitute for the emotional and relational functions of a marital relationship, depriving couples of communication and interactions. Long-term communication deficiencies due to a mobile phone or Internet addiction erodes the intimate relationship between a husband and wife and triggers a marriage crisis. Based on these factors, we propose the fourth hypothesis:

**Hypothesis** **4.** *Internet use reduces marital stability through a decrease in communication between a husband and wife.*

## 3. Study Design, Materials, and Methods

### 3.1. Study Design

As discussed above, the Internet may affect the risk of divorce. If the inference at the individual level holds true, we can reasonably anticipate a positive association between the IPR and CDRs at the provincial level. Therefore, we employed an individual-level dataset to investigate the causal effect of the Internet and its mechanisms on divorce risk, and then used provincial datasets to further verify the conclusions of the micro research.

### 3.2. Materials and Methods of Individual-Level Analysis

#### 3.2.1. Methods

The Cox regression model is widely used in the medical sphere to predict patients’ survival time as the result of one or more predictive variables [49]. It allows us to examine how specific factors affect the incidence of specific events (e.g., infection, death, divorce) at specific points in time. We adopted the Cox regression model to explore the effect of Internet use on divorce risk. The outcome variable *Divorce* was an indicator variable; if an individual was divorced, *Divorce* = 1; otherwise, *Divorce* = 0. The survival time *T* was equal to the duration (in years) of a marriage. The predicting factors of interest were Internet access and Internet usage behaviors. If an individual used mobile Internet, *Mint* = 1; otherwise, *Mint* = 0. *Intimp*, which ranged from 1 to 3, measured the importance of respondents’ access to information through the Internet. *Intchat*, *Intmet*, and *Intfri*, which ranged from 1 to 3, measured the frequency of respondents “chatting, meeting, and becoming friends in reality” with online friends, respectively.

*Gender* was a dummy variable equal to 1 for male and 0 for female. Residential location (*Urban*) was a dummy variable that took the value of 1 for urban residents and the value of 0 for others. Regional location (*Region*) was coded as 1–4 and denoted Eastern, Central, Western, and Northeast China, respectively. The absolute value of the age difference (*Difage*) and education difference (*Difeduc*) between a husband and wife were also used, as previous studies have shown that the age and education gap may affect marital stability. As a typical marriage-specific investment, children were considered to be associated with marital stability; thus, the number of children (*Childn*) was included. The economic status (*Ecosta*) and social status (*Socsta*) represented the self-reported relative economic and social status and were coded as 1–3.

#### 3.2.2. Materials of Individual-Level Analysis

The micro data originated from the datasets of the CFPS, released in 2010 and 2016. The CFPS is a nationally representative survey conducted by the China Social Science Research Center of Peking University, which aims to reflect the social and economic changes in China by tracking and collecting data from individual, family, and community levels (http://www.isss.pku.edu.cn/cfps/index.htm (accessed on 25 December 2022)). This dataset surveys, in detail, the marriage history of individuals as well as Internet access and usage behaviors, which provided appropriate data for this study. According to research requirements, missing data and outliers were deleted, and the variables used in the empirical study were recoded. With regard to first marriages, couples may confront different levels of divorce risk as compared to those of remarried couples; thus, we deleted the data from remarried couples. Singles face no risk of divorce and were, therefore, deleted. After this data cleaning and coding, we ultimately obtained 16,456 pieces of available core data. Table 1 presents the summary descriptive statistics.

### 3.3. Materials and Methods of Provincial Analysis

#### 3.3.1. Methods of Provincial Analysis

The macro study employed the panel data regression method to investigate the impact of the IPR on the divorce rates. Specifically, the benchmark model is shown in Equation (1):

(1) $$Dvr_{it}=\beta_{0}+\beta_{1}(Intp_{it})+\sum_{j=2}^{n}\beta_{j}X_{it}+\mu_{i}+\lambda_{t}+\varepsilon_{it}$$

where the subscript *i* = 1, 2, …, 31 represents each of the 31 provinces, and *t* = 1, 2, …, 11 denotes each specific year from 2005 to 2016. The dependent variable $Dvr_{it}$ is the CDRs of province *i* in year *t*. The core explanatory variable $Intp_{it}$ is the broadband IPR of province *i* in year *t*. $X_{it}$ represents a group of control variables. $\beta_{0}$ is a constant term, and $\beta_{1}$ represents the coefficient of the independent variable of interest. $\beta_{j}(j\geq 2)$ represents the coefficients of the control variables. $\mu_{i}$ and $\lambda_{t}$ represent provincial and year fixed effects, respectively. $\varepsilon_{it}$ is a random disturbance term. The control variables included the sex ratio (*Sexr*), urbanization rate (*Ubnr*), real per capita GDP (*Pinc*), average of education (*Edu*), and total dependency ratio (*Pdr*). The detailed definitions of variables can be found in Table 2.

#### 3.3.2. Datasets of Provincial Analysis

The provincial data came mainly from various statistics yearbooks of China from 2006 to 2017. CDRs were collected from the *China Civil Affairs’ Statistical Yearbook*. The per capita education, per capita real income, and per capita dependency ratio were sourced from the *China Population and Employment Statistics Yearbook*. The provincial IPR was collected from the *Statistical Report on the Development of Internet in China* released by the CNNIC (http://www.cnnic.net.cn/hlwfzyj/hlwxzbg/ (accessed on 25 December 2022)). Other indicators were from the *China Statistical Yearbook*. As the provincial variables showed a skewed distribution, all variables were logarithmized. Table 2 also shows the summary statistics for the provincial data.

**Figure 2:**
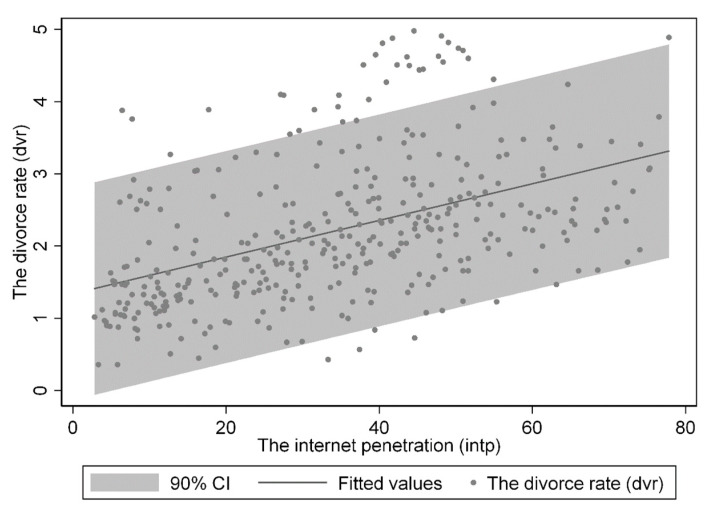
Scatter plot of CDRs and IPR.

Figure 2 depicts the relationship between the CDRs and the IPR for the 31 provinces in China. This scatter plot shows a significant positive correlation between the CDRs and the IPR. Moreover, most scatters fell into the 90-percent confidence interval of the fitting line. Nevertheless, this preliminary evidence merely suggests a positive association between the CDRs and the IPR; further analysis is needed to support causal inference.

## 4. Microscopic Analysis: The Impact of Internet Use on Divorce Risk

### 4.1. Estimated Results of Cox Regression

**Figure 3:**
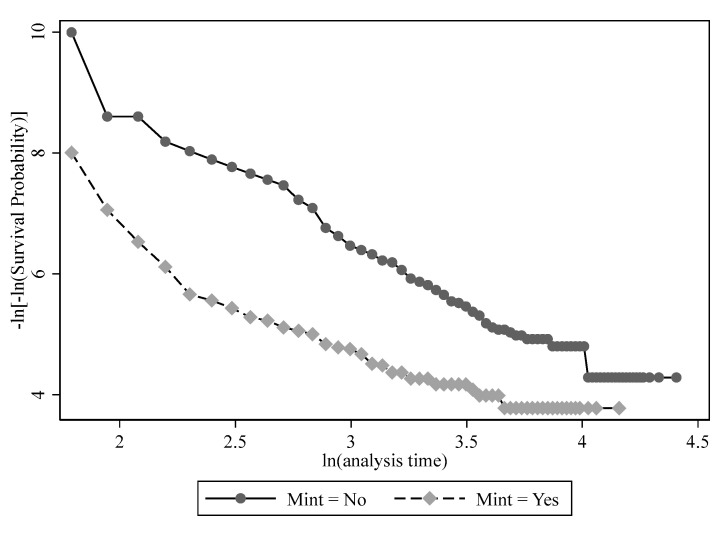
Assessment of proportional hazards assumption for Internet access.

The proportional risk assumption is the precondition of the Cox regression model. The assessment of the proportional hazards (PH) assumption of Internet access is shown in Figure 3. As can be seen, the line of *Mint* = yes is almost always parallel to the line of *Mint* = no, without any point of intersection at any time point; thus, the PH hypothesis holds. The other core explanatory variables, including the importance of the Internet in obtaining information, all passed the PH test. The graphs are not reported due to space restriction.

The Cox regression estimation results are shown in Table 3. According to Model (1), mobile Internet access significantly increases the divorce risk of individuals, after filtering out the confounds of a variety of covariates. In other words, compared with those who never use mobile Internet, mobile Internet users have a higher divorce risk.

According to Model (2), the importance of the Internet for obtaining information is positively associated with a higher divorce risk. The stronger the dependence of the respondents on the Internet when searching for information, the higher the possibility of marriage breakdown. As noted, Internet penetration has promoted the dissemination of modern marriage notions and accelerated the collapse of traditional marital values. People with a modern marriage concept no longer consider divorce to be shameful. Therefore, they bear less psychological and public desirability pressure in terms of dissolving their troubled marriage and, thus, are bolder in terminating their unhappy marriage. This result provides preliminary evidence for Hypothesis 1.

In keeping with Model (3), there was a positive association between the divorce risk and the frequency of chatting with online friends. The Internet is an efficient and low-cost channel to find a partner, which greatly reduces the search cost of potential partners and increases the risk of divorce. The above analysis provides evidence for Hypothesis 2.

Model (4) shows a positive correlation between the frequency of meeting with online friends in real life and the divorce risk. In the case of divorce, having a larger scale of online friends expands, to some extent, the range of available and suitable alternative partners. The positive influence of online friends on divorce risk is consistent with the notion of an alternative partner. The above analysis suggests the rationality of Hypothesis 3.

Model (5) indicates that Internet usage intensity is significantly positively correlated with divorce risk. The extensive use of the Internet impinges on the time that couples should spend on emotional support. This leads to a sharp decline in marital happiness and satisfaction and to irreconcilable differences, ultimately resulting in the breakdown of the marriage. This analysis provides preliminary evidence for Hypothesis 4.

The coefficients of the covariates also revealed some interesting facts. Male respondents were confronted with a higher divorce risk than their female counterparts. A larger age difference between a husband and wife was a statistically significant risk factor for marriage. A negative correlation was found between the number of children and the divorce risk. As noted by Becker (1974), couples tend to invest more intensively in their minor children, which helps to improve the couple’s identification with the family and reduce the possibility of a marital split. Self-reported social status is a significant inhibiting factor for marriage dissolution. The higher the social status of the respondents, the less economic and social pressure the couples may face, thereby reducing the possibility of conflict between couples. This appears to be consistent with the old Chinese saying, “nothing goes well for a destitute couple.”

Table 4 presents the estimation results by region and gender, showing a significant positive correlation between Internet use and divorce risk across regions and genders. The magnitude of the impact, however, showed regional and gender heterogeneity. The magnitude was the strongest in Central China, followed by the Eastern and Western regions, and it was the lowest in the Northeast region. The Eastern provinces have undergone rapid economic development and comprehensive social reforms. Therefore, modern marriage notions have become deeply rooted. Modern marriage notions have received limited impact from Internet penetration in the Eastern region. Urbanization and industrialization have been slower in the Central region, and the concept of traditional marriage remains popular. Thus, the rapid spread of the Internet in Central China has accelerated the disintegration of traditional marriage concepts and ultimately increased the regional divorce rates. The Western region has experienced the lowest level of economic development, with relatively low Internet penetration and divorce rates. The divorce rates in Northeastern China are the highest in the country. There are several unique reasons for this phenomenon, including the high urbanization rate, female education, the region’s immigrant culture, and the high proportion of a minority population. The Internet is just one of the drivers that promote this significant marriage instability.

The coefficient of Internet use for women is larger than that for men, which indicates that females who use the Internet experience a higher divorce risk than their male counterparts. Access to Internet information has changed women’s traditional notion of marriage, making women more able to end an unhappy marriage than ever before. From this point of view, information access through the Internet gives women the initiative to decide their own marital status. To some extent, this can be seen as a sign of women’s rising marital and social status.

### 4.2. Robustness Checks

#### 4.2.1. Changing the Sample–Time Bandwidth

The rapid acceptance and use of the Internet started early in the 21st century in China. As the whole sample pooled all first-married couples, including those who married before 2000, the reliability of the results using a full sample estimate was partially weakened. To overcome the above-mentioned disadvantage, couples who married after 2000 were extracted to form a subsample. The estimated results of this subsample are shown in Table 5. The coefficients of the core explanatory variables were consistent with the whole-sample estimation. Internet access and Internet usage behavior were positively correlated with a high risk of divorce.

#### 4.2.2. Propensity Score-Matching Method

**Figure 4:**
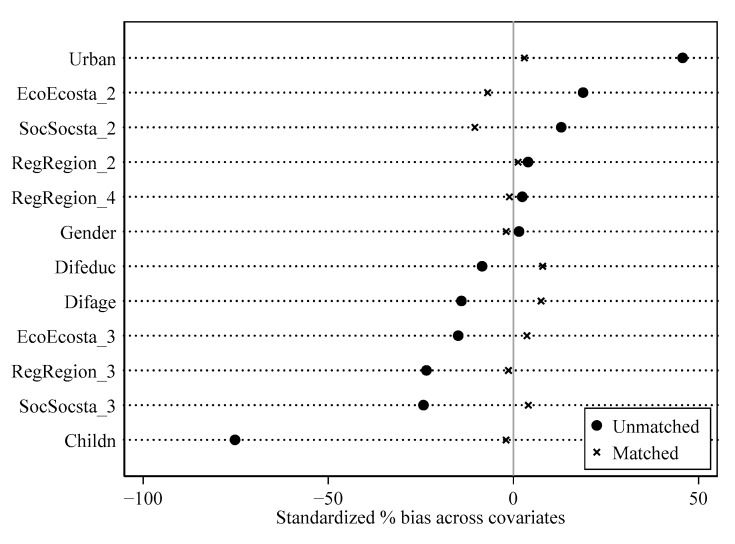
Balancing test.

Propensity score matching was used to further control the possible selection bias caused by the non-randomness of sampling. Table 6 provides the estimation results under four matching methods: nearest-neighbor matching, radius matching, kernel matching, and local linear regression matching. The balance tests were all satisfied for the four matching methods. Figure 4 shows that, after performing nearest-neighbor matching, there was no systematic difference between the control group and the treatment group. Due to space constraints, the balance tests of the other three matching methods are not reported. The results showed that, after tackling selective bias, the average impact of Internet access on the divorce risk was significantly positive. The estimated coefficients under the four methods were very close, which further verified the robustness of the estimated results.

### 4.3. The Impact Mechanism of Internet Use on Divorce Risk

Based on the mediating effect model, we studied the mechanisms of the impact of the Internet on divorce risk. Generally speaking, a mediation effect is significant when (1) the independent variable (IV) has a significant impact on the mediating variable (*MV*), (2) the IV significantly affects the dependent variable (*DV*) in the absence of the *MV*, (3) the mediator has a significant unique effect on the *DV*, and (4) the effect of the IV on the *DV* shrinks upon the addition of the MV to the model [50]. By successively estimating Models (2), (3), and (4), we calculated the mediation effect.

(2) $$Divorce_{it}=\alpha_{0}+c\times Mint_{it}+\eta \times CV_{it}+\varepsilon_{it}$$

(3) $$MV_{it}=\beta_{0}+a\times Mint_{it}+\lambda \times CV_{it}+\varepsilon_{it}$$

(4) $$Divorce_{it}=\gamma_{0}+c^{\prime }Mint_{it}+bM_{it}+\mu CV_{it}+\varepsilon_{it}$$

The mediation effect model estimation results are shown in Table 7. Models (1) to (3), Models (4) and (5), Models (6) and (7), and Models (8) and (9) tested the mediating effect of the importance of Internet information acquisition, the frequency of chatting with online friends, the frequency of meeting with online friends, and the intensity of Internet use, respectively. Model (1) showed that Internet access had a significant positive impact on divorce risk. Model (2) demonstrated a positive correlation between Internet access and the importance of Internet information acquisition. Model (3) showed the estimation results of divorce risk in relation to Internet access and the importance of Internet information acquisition. The coefficient of Internet access was reduced by half compared with Model (1). The Sobel–Goodman test indicated a significant mediation effect. The mediation effect accounted for 0.4874 of the total effect, which means that 48.74% of the total effect was attributed to the mediation of the importance of Internet information acquisition. Similarly, the frequency of chatting with online friends, the frequency of meeting with online friends, and the intensity of Internet use all played a mediating role in the impact of Internet access on divorce risk. Specifically, the mediation effects accounted for 35.70%, 8.06%, and 44.39%, respectively. The above estimated results provide empirical support for the four hypotheses.

## 5. Impact of Internet Penetration Rate on Crude Divorce Rates in China

### 5.1. Estimated Results of Baseline Model

The micro data study revealed that Internet access increases divorce risk. We may not, however, have included all risk factors in the Cox regression model due to data availability. These unobservable factors may have caused the estimation bias of the omitted variables. Fortunately, the provincial panel data had the advantage of controlling for unobservable factors by adding the provincial fixed effect. Models (1)–(6) in Table 8 successively present the estimated results with the addition of more control variables. The coefficients of interest in the six models were all significantly positive. It can be concluded that the pervasiveness of the Internet is one of the factors that has increased divorce rates.

### 5.2. Estimated Results of the Impact of IPR on CDRs by Region

The sub-regional estimation results are reported in Table 9. The results suggest that the IPR had a significant positive effect on the CDRs in all regions. The magnitude of the impact, however, showed regional heterogeneity. Specifically, the magnitude was highest in the Central region, followed by the Western region, with the Eastern region as third, and the Northeastern region as last. Table 4 presents the heterogeneous impact of mobile Internet access on the personal divorce risk on a micro level. In contrast, Table 9 provides the estimated results of the heterogeneous impact of Internet popularization on divorce rates at the provincial level. It is striking that the coefficients of the core explanatory variables in these four regions were consistent at the individual- and provincial-level studies. The conclusions of the micro research were verified by the provincial-level research.

### 5.3. Endogenous Treatment

The estimation results of the least squares method may have endogeneity problems. The basic assumption in the estimation is that the Internet has a significant impact on divorce rates, but not vice versa. The increase in divorce rates, however, may affect the popularity of online dating services and increase the number of Internet users. The above two-way causality leads to endogeneity problems.

One way to address endogeneity is the instrumental variable method. To this end, we needed to find qualified instrumental variables for the IPR. The qualified instrumental variables needed to meet the relevance condition and exclusion restriction; namely, the instrumental variables should be highly correlated with the IPR, but cannot be directly correlated with CDRs at the same time. As the length of long-distance optical cable lines (*Cabl*) and the capacity of mobile telephone exchanges (*Texc*) are the infrastructure of the mobile Internet, and basically will not directly affect CDRs, they qualified as instrumental variables. Table 10 shows the instrumental estimation results. The under-identification test, weak identification test, and Sargan test of instrumental variables were all satisfied. After addressing the possible endogeneity problems associated with instrumental variables, the positive impact of the IPR on the CDRs still held.

## 6. Discussion and Conclusions

In recent years, with the rapidly growing use of the Internet, CDRs have risen sharply. Prior studies have extensively explored the influencing factors of the surge in the CDRs from economic development and social reform perspectives, but limited attention has been paid to the impact of technological factors, such as the Internet, on the divorce risk, especially in a populous, developing country such as China, which has historically been influenced by Confucian culture. Countries influenced by Confucian culture usually have high marriage stability, as divorce is considered a stigma. Access to the Internet, with its profound implications associated with information acquisition and interpersonal modes, has had a significant impact on traditional marriage notions and marriage stability. In the context of China, this paper employs individual-level data and provincial panel data to investigate the impact of the Internet on marital stability at the micro and provincial levels.

Following the research of Valenzuela et al. (2014) and Zheng et al. (2019), this paper investigated the effect of Internet access on marital stability by using a nationally representative sample of first marriages. We then employed the provincial panel data to explore the association between the IPR and CDRs. The robustness of our conclusions was confirmed in several ways. For the micro research, we explored the relationship between the Internet and divorce risk across time periods, regions, and genders. We also addressed the potential self-selection problem through the use of the propensity score-matching method. For the macro studies, we used the instrumental variable method to verify the causal inference of the positive impact of the IPR on CDRs. The most important finding was that there is a significant positive correlation between Internet access and divorce risk. Specifically, the micro studies showed that mobile Internet access and Internet usage behaviors significantly increased the divorce risk. Correspondingly, the macro studies illustrated that an increase in the IPR results in a rise in CDRs after controlling for the influence of covariates and addressing potential endogeneity problems. The estimated results from the provincial perspective echoed the conclusions of the micro research.

To obtain a comprehensive understanding of the impact of information access through the Internet on divorce risk, we adopted a mediation effect model to identify four channels through which Internet access may affect divorce risk. First, the popularization of the Internet has promoted the dissemination of modern marriage notions and affected people’s concepts of marriage, thereby increasing the risk of divorce. Second, the Internet is an efficient and low-cost channel for finding a marriage partner, which greatly reduces the cost of searching for potential partners and increases the risk of divorce. Third, the Internet provides convenience in terms of making friends online. Face-to-face meetings with online friends, especially those of the opposite sex, can lead to the suspicion of infidelity, which can significantly aggravate the conflicts between marriage partners and even lead to the breakdown of the marriage. Finally, the Internet provides a variety of interesting multimedia entertainment content, and the heavy use of Internet by couples can impinge on the time that they spend on emotional communication. Consequently, marital happiness and satisfaction will drop sharply, leading to irreconcilable differences and, eventually, to the breakdown of the marriage.

This study also made a contribution to theory. To the best of our knowledge, this paper is the first to directly examine the relationship between Internet access and divorce risk in the context of Asian culture. From the perspective of information access through the Internet, this paper provides a new explanation for the rise in CDRs in China, which further enriches the research on marital stability and the impact of technological developments on human relationships and behavior. The findings of this study also have several important implications for public policymaking. Internet technology has been widely regarded as one of the driving forces of economic development. Policymakers have paid limited attention, however, to the potential impact of information technology on social interactions, marriage stability, and family relations. This study shows that the penetration of the Internet has an important impact on marriage stability. Therefore, with further popularization and the application of information technology, developing countries and regions may also witness a rise in divorce rates. Public policymakers should not neglect the impact of Internet penetration on marriage disruption.

At the same time, it is worth noting that policymakers must realize that divorce does not necessarily bring about a decline in welfare. If Internet use accelerates the early end of unfortunate marriages by improving information access, it will not bring welfare losses. Instead, a rational choice based on sufficient information by both spouses can improve their welfare. The cooling-off period is a policy designed to curb the rising divorce rate. The basic assumption of this policy is that the increasing divorce is due to individuals not being rational enough and needing more information before making decisions. Our research found, however, that the positive impact of the Internet on the divorce rate is through the information acquisition effect. In modern society, the Internet provides information that helps people overcome the fear of divorce and reduces the potential cost of divorce. In addition, the Internet helps people find a more suitable partner after divorce. The use of the Internet may accelerate the early termination of a problematic marriage, which is a more rational choice made by the couple based on more abundant information and wider alternative marital partners and which has an improvement effect on the welfare of both members of the couple. Therefore, a mandatory cooling-off period may hinder people from ending unhappy marriages and deprive couples in unhappy marriages of the possibility of improving their welfare through divorce.

Further empirical exploration is required to refine and improve this empirical work. Due to the availability of datasets, it is necessary to further investigate the policy effect and welfare effect of the cooling-off period policy by using updated datasets in the future. The use of up-to-date datasets can enhance the timeliness and effectiveness of research. In addition, more work is needed to further explore the mechanism by which Internet use affects marital stability. Furthermore, with same-sex marriages increasing worldwide [51,52], the field of research on marital stability has generated a series of worthy research issues. For example, what are the driving factors for the formation and breakdown of same-sex marriages? How does Internet use affect the stability of same-sex marriage relationships? What impact will the legalization of same-sex marriage have on the stability of marriage?

## Figures and Tables

**Table 1:** Summary statistics of key variables for individual-level data (*N* = 16,456).

Variable	Definition	Mean	SD	Min.	Max.
*Divorce*	Marital status	0.010	0.101	0	1
*Mint*	Use of mobile	0.280	0.449	0	1
*Intimp*	Importance of the Internet in obtaining information	1.511	0.692	1	3
*Intchat*	Frequency of chatting with online friends	1.052	0.232	1	3
*Intmet*	Frequency of meeting online friends	1.004	0.069	1	3
*Intime*	Internet usage intensity	1.416	0.787	1	3
*T*	Years of marriage	29.17	12.85	6	82
*Gender*	Gender	0.488	0.500	0	1
*Urban*	Residential location	0.424	0.494	0	1
*Difage*	Age difference between husband and wife	2.597	2.534	0	43
*Difeduc*	Education difference between husband and wife	0.802	0.845	0	5
*Childn*	Number of children	1.999	1.044	0	10
*Ecosta*	Economic status	1.820	0.471	1	3
*Socsta*	Social status	1.960	0.453	1	3
*Region*	Regional location	2.253	1.061	1	4

**Table 2:** Summary statistics of key variables for provincial data (*N* = 372).

Variable	Definition	Mean	SD	Min.	Max.
*Dvr*	Crude divorce rate (%)	2.205	1.004	0.360	4.980
*Dvr2*	Ratio of divorces to marriages (%)	25.715	11.175	6.633	66.395
*Intp*	Internet penetration rate (%)	34.059	18.613	2.800	77.800
*Sexr*	Sex ratio (%)	104.831	3.658	94.650	120.430
*Ubnr*	Urbanization rate (%)	51.453	14.724	20.850	89.600
*Pinc*	Real per capita GDP (unit)	19,405.830	11,095.974	5394.000	54,434.502
*Edu*	Average education	8.638	1.198	3.740	12.300
*Pdr*	Total dependency ratio	37.648	7.789	19.270	62.980
*Cabl*	Length of long-distance optical cable lines (Km)	4832.268	4013.049	66.000	22,025.800
*Texc*	Capacity of mobile telephone exchanges (10,000 subscribers)	26,993.206	14,291.045	642.300	77,483.000

**Table 3:** Estimated results for the effect of Internet use on divorce risk (*N* = 16,456).

Model	(1)	(2)	(3)	(4)	(5)
*Mint*	1.435 ***				
	(0.169)				
*Intimp*		0.881 ***			
		(0.101)			
*Intchat*			1.568 ***		
			(0.179)		
*Intmet*				1.929 ***	
				(0.361)	
*Intime*					0.689 ***
					(0.087)
*Gender*	0.013 *	0.013 *	0.010	0.012 *	0.013 *
	(0.007)	(0.007)	(0.007)	(0.007)	(0.007)
*Urban*	−0.001	0.005	0.009	0.010	0.001
	(0.008)	(0.008)	(0.008)	(0.008)	(0.008)
*Difage*	0.004 ***	0.004 ***	0.003 ***	0.003 ***	0.004 ***
	(0.001)	(0.001)	(0.001)	(0.001)	(0.001)
*Difeduc*	−0.001	−0.001	−0.001	−0.001	−0.001
	(0.004)	(0.004)	(0.004)	(0.004)	(0.004)
*Childn*	−0.041 ***	−0.041 ***	−0.046 ***	−0.046 ***	−0.042 ***
	(0.005)	(0.005)	(0.005)	(0.005)	(0.005)
*Ecosta = 2*	−0.013	−0.015 *	−0.010	−0.010	−0.013
	(0.009)	(0.009)	(0.009)	(0.009)	(0.009)
*Ecosta = 3*	−0.012	−0.018	−0.015	−0.014	−0.013
	(0.022)	(0.022)	(0.022)	(0.022)	(0.022)
*Socsta = 2*	−0.016 *	−0.017 *	−0.016 *	−0.017 *	−0.017 *
	(0.010)	(0.010)	(0.010)	(0.010)	(0.010)
*Socsta = 3*	−0.022	−0.027	−0.026	−0.028	−0.023
	(0.018)	(0.018)	(0.018)	(0.018)	(0.018)
Regional effect	Yes	Yes	Yes	Yes	Yes
*LR*	242.505 ***	241.952 ***	218.097 ***	183.967 ***	228.722 ***

Notes: Standard errors are reported in parentheses. * and *** represent *p* < 0.10 and *p* < 0.01, respectively.

**Table 4:** Estimated results for the effect of Internet use on divorce risk by region and gender.

Model	(1)	(2)	(3)	(4)	(5)	(6)
Subsample	Eastern	Central	Western	Northeastern	Female	Male
*Mint*	1.484 ***	1.641 ***	1.369 ***	1.261 ***	1.493 ***	1.404 ***
	(0.331)	(0.372)	(0.357)	(0.325)	(0.261)	(0.222)
Control variable	Yes	Yes	Yes	Yes	Yes	Yes
*N*	5283	4146	4607	2420	8422	8034
*LR*	65.137 ***	78.620 ***	53.623 ***	48.176 ***	109.961 ***	144.868 ***

Notes: Standard errors are reported in parentheses. *** represents *p* < 0.01.

**Table 5:** Estimated results for Internet usage on divorce risk (*N* = 3047).

Model	(1)	(2)	(3)	(4)	(5)
*Mint*	0.786 **				
	(0.315)				
*Intimp*		0.386 **			
		(0.180)			
*Intchat*			0.737 ***		
			(0.260)		
*Intmet*				1.568 ***	
				(0.498)	
*Intime*					0.470 ***
					(0.143)
Control variable	Yes	Yes	Yes	Yes	Yes
*LR*	49.018 ***	46.659 ***	48.979 ***	48.291 ***	53.590 ***

Notes: Standard errors are reported in parentheses. ** and *** represent *p* < 0.05, and *p* < 0.01, respectively.

**Table 6:** PSM analysis of the impact of Internet use on divorce risk (*N* = 16,456).

Method	Nearest-Neighbor	Radius	Kernel	Local Linear Regression
ATT	0.009 ***	0.008 ***	0.008 ***	0.008 **
	(0.002)	(0.002)	(0.002)	(0.002)
Control variables	Yes	Yes	Yes	Yes
Regional effect	Yes	Yes	Yes	Yes
Treated	4614	4614	4614	4614
Untreated	11,842	11,842	11,842	11,842

Notes: Standard errors are reported in parentheses. ATT = average treatment effect on treatment. ** and *** represent *p* < 0.05 and *p* < 0.01, respectively.

**Table 7:** Mediation effect results of Internet use on divorce risk (*N* = 16,456).

Model	(1)	(2)	(3)	(4)	(5)	(6)	(7)	(8)	(9)
Mediation variable	*Intimp*			*Intchat*		*Intmet*		*Intime*	
Dependent variable	*Divorce*	*Intimp*	*Divorce*	*Intchat*	*Divorce*	*Intmet*	*Divorce*	*Intime*	*Divorce*
*Mint*	0.008 ***	0.944 ***	0.004 *	0.179 ***	0.005 ***	0.014 ***	0.008 ***	1.333 ***	0.005 *
	(0.002)	(0.010)	(0.002)	(0.004)	(0.002)	(0.001)	(0.002)	(0.009)	(0.003)
*Intimp*			0.004 ***						
			(0.002)						
*Intchat*					0.017 ***				
					(0.004)				
*Intmet*							0.049 ***		
							(0.011)		
*Intime*									0.003 *
									(0.002)
Control variables	Yes	Yes	Yes	Yes	Yes	Yes	Yes	Yes	Yes
Constant	Yes	Yes	Yes	Yes	Yes	Yes	Yes	Yes	Yes
Adj. *R*^2^	0.009	0.436	0.009	0.121	0.010	0.009	0.010	0.614	0.009
*F*	12.160	980.059	11.896	174.800	12.876	12.269	12.651	2016.300	11.514
Sobel–Goodman	2.895 ***			4.662 ***		4.016 ***		1.758 *	
Mediating effect	48.74%			35.70%		8.06%		44.39%	

Notes: Standard errors are reported in parentheses. * and *** represent *p* < 0.10 and *p* < 0.01, respectively.

**Table 8:** Estimated results of IPR on the CDRs (*N* = 372).

Model	(1)	(2)	(3)	(4)	(5)	(6)
*Intp*	0.357 ***	0.331 ***	0.337 ***	0.321 ***	0.367 ***	0.366 ***
	(0.011)	(0.018)	(0.019)	(0.019)	(0.020)	(0.020)
*Aedu*		0.326 *	0.348 **	0.343 **	0.333 **	0.272
		(0.175)	(0.176)	(0.174)	(0.165)	(0.171)
*Hscl*			0.233	0.395 *	0.257	0.233
			(0.209)	(0.211)	(0.202)	(0.203)
*Popu*				0.655 ***	0.299	0.213
				(0.188)	(0.189)	(0.198)
*Rgdp*					−0.363 ***	−0.356 ***
					(0.061)	(0.061)
*Sexr*						0.473
						(0.337)
Constant	Yes	Yes	Yes	Yes	Yes	Yes
Provincial fixed effect	Yes	Yes	Yes	Yes	Yes	Yes
Year fixed effect	Yes	Yes	Yes	Yes	Yes	Yes
Adj. *R*^2^	0.730	0.732	0.732	0.741	0.765	0.766
*F*	1035.859	523.406	349.597	273.869	248.857	208.303

Notes: Standard errors are reported in parentheses. *, **, and *** represent *p* < 0.10, *p* < 0.05, and *p* < 0.01, respectively.

**Table 9:** Estimated results for the effects of IPR on CDRs by region.

Model	(1)	(2)	(3)	(4)
Region	Eastern	Central	Western	Northeastern
*Intp*	0.331 ***	0.404 ***	0.389 ***	0.273 ***
	(0.043)	(0.044)	(0.048)	(0.035)
Control variable	Yes	Yes	Yes	Yes
Provincial fixed effect	Yes	Yes	Yes	Yes
Year fixed effect	Yes	Yes	Yes	Yes
*F*	75.269	100.767	52.230	110.083
Adj. *R*^2^	0.769	0.877	0.715	0.949
*N*	132	84	120	36

Notes: Standard errors are reported in parentheses. *** represents *p* < 0.01.

**Table 10:** Instrumental regression results for the effects of IPR on CDRs (*N* = 372).

Model	(1)	(2)
Dependent variable	*Intp*	*Dvr*
*Intp*		0.396 ***
		(0.023)
*Cabl*	0.946 ***	
	(0.034)	
*Texc*	−0.011	
	(0.067)	
Control variable	Yes	Yes
Adj. *R*^2^	0.269	0.787
*F*	621.080	199.400
Under-identification test	252.610 ***	
Weak identification test	477.269	
Stock–Yogo weak test, critical values	19.93(10%)	
Sargan statistic	0.909	

Notes: Standard errors are reported in parentheses. *** represents *p* < 0.01.

## Data Availability

The data of this study are available from the corresponding author upon reasonable request.
